# PAI‐1, a target gene of miR‐143, regulates invasion and metastasis by upregulating MMP‐13 expression of human osteosarcoma

**DOI:** 10.1002/cam4.651

**Published:** 2016-01-28

**Authors:** Mio Hirahata, Mitsuhiko Osaki, Yusuke Kanda, Yui Sugimoto, Yusuke Yoshioka, Nobuyoshi Kosaka, Fumitaka Takeshita, Tomohiro Fujiwara, Akira Kawai, Hisao Ito, Takahiro Ochiya, Futoshi Okada

**Affiliations:** ^1^ Division of Pathological BiochemistryDepartment of Biomedical SciencesFaculty of MedicineTottori University86 Nishi‐choYonagoTottori683‐8503Japan; ^2^ Chromosome Engineering Research CenterTottori University86 Nishi‐choYonagoTottori683‐8503Japan; ^3^ Division of Molecular and Cellular MedicineNational Cancer Center Research InstituteNational Cancer Center Hospital5‐1‐1 TsukijiChuo‐kuTokyo104‐0045Japan; ^4^ Orthopedics DivisionNational Cancer Center Hospital5‐1‐1 TsukijiChuo‐kuTokyo104‐0045Japan

**Keywords:** Osteosarcoma, metastasis, miR‐143, PAI‐1, MMP‐13

## Abstract

Despite recent improvements in the therapy for osteosarcoma, 30–40% of osteosarcoma patients die of this disease, mainly due to its lung metastasis. We have previously reported that intravenous injection of miR‐143 significantly suppresses lung metastasis of human osteosarcoma cells (143B) in a mouse model. In this study, we examined the biological role and mechanism of miR‐143 in the metastasis of human osteosarcoma cells. We identified plasminogen activator inhibitor‐1 (PAI‐1) as a direct target gene of miR‐143. To determine the role of PAI‐1 in human osteosarcoma cells, siRNA was transfected into 143B cells for knockdown of PAI‐1 expression. An in vitro study showed that downregulation of PAI‐1 suppressed cell invasion activity, but not proliferation. Moreover, injection of PAI‐1 siRNA into a primary lesion in the osteosarcoma mouse model inhibited lung metastasis compared to control siRNA‐injected mice, without influencing the proliferative activity of the tumor cells. Subsequent examination using 143B cells revealed that knockdown of PAI‐1 expression resulted in downregulation of the expression and secretion of matrix metalloproteinase‐13 (MMP‐13), which is also a target gene of miR‐143 and a proteolytic enzyme that regulates tumor‐induced osteolysis. Immunohistochemical analysis using clinical samples showed that higher miR‐143 expressing cases showed poor expression of PAI‐1 in the primary tumor cells. All such cases belonged to the lung metastasis‐negative group. Moreover, the frequency of lung metastasis‐positive cases was significantly higher in PAI‐1 and MMP‐13 double‐positive cases than in PAI‐1 or MMP‐13 single‐positive or double‐negative cases (*P *<* *0.05). These results indicated that PAI‐1, a target gene of miR‐143, regulates invasion and lung metastasis via enhancement of MMP‐13 expression and secretion in human osteosarcoma cells, suggesting that these molecules could be potential therapeutic target genes for preventing lung metastasis in osteosarcoma patients.

## Introduction

Osteosarcoma is a primary bone tumor that mainly affects young children and adolescents. Despite recent advances in multimodality treatments that consist of adjuvant chemotherapy and surgical‐wide resection, pulmonary metastasis occurs in ~40–50% of the patients 1. In such cases, the overall 5‐year survival rate is only 28%, despite multidisciplinary therapy 2. Thus, it is important to suppress lung metastasis in osteosarcoma for improvement of prognosis.

MicroRNA (miRNA) belongs to a class of endogenously expressed, non‐coding small RNA, and contains about 22 nucleotides. Based on the database miRBase release 16.0, >1000 human miRNAs have been registered and a large number of these are evolutionarily conserved 3. miRNA regulates the expression of protein‐coding genes at the posttranscriptional level through mRNA degradation or suppression of peptide chain elongation 4. miRNA is predicted to regulate the expression of at least 30% of all genes 5. A growing amount of evidence suggests that deregulation of miRNA may contribute to many types of human diseases, including cancer. Errors in the expression of miRNA have been observed in various types of cancers 6, 7 and are also associated with the clinical outcome of cancer patients 8, 9. Consistently, miRNA has been implicated in the regulation of various cellular processes that are often deregulated during tumor development and progression, 10, 11, 12, suggesting that miRNA might be a target for cancer therapy.

Recently, our group reported that intravenous injection of miR‐143 significantly suppressed the lung metastasis of human osteosarcoma cells (143B) in a mouse model. Moreover, we showed that plasminogen activator inhibitor 1 (PAI‐1) was a candidate target gene of miR‐143 in 143B cells by LAMP (labeled miRNA pull down) assay and Ago2 IP with miR‐143 target database 13. PAI‐1 is known to inhibit the plasminogen activator enzyme and to be involved in coagulation 14. On the other hand, PAI‐1 is also related to carcinogenesis. PAI‐1 is upregulated in malignant cancers such as breast cancer 15 and ovarian carcinoma 16. Moreover, PAI‐1 is related to malignancies by influencing tumor migration, invasion, angiogenesis 17, 18, 19, 20, and metastasis 21. However, the mechanism by which PAI‐1 regulates lung metastasis of osteosarcoma cells has been unclear. The purpose of this study was to identify the role of miR‐143 in the invasion and metastasis of human osteosarcoma cells, with a special focus on PAI‐1 expression.

## Materials and Methods

### Cell culture and transfection

The human osteosarcoma cell line, 143B, was obtained from the American Type Culture Collection and was maintained in Dulbecco's modified Eagle's medium containing 10% heat‐inactivated fetal bovine serum. The 143B cells were transfected with a complex of the pLuc‐Neo plasmid DNA (143B‐Luc) as described previously 13. Synthetic pre‐hsa‐miR‐143 (Ambion, Austin, TX), PAI‐1 siRNA: 5′‐AAGCACAACUCCCUUAAGGUCTT‐3′ 22, 23, or control small RNA (miR‐NC1; Ambion, Mission siRNA Universal negative control; Sigma, St Louis, MO) were each transfected into 143B cells at a concentration of 30 nmol/L per 2.5 × 10^5^ cells in a 6 cm dish using DharmaFECT (GE Healthcare, Buckinghamshire, England).

### Clinical samples

Fifty‐two biopsy samples of human osteosarcomas, which did not have metastasis in the lung and other organs at first diagnosis, were obtained from the National Cancer Center Hospital. All the materials were obtained with written informed consent, and the procedures were approved by the institutional review board (approval number: 2312).

### Western blotting

Cells were washed in phosphate buffered saline (PBS) and solubilized in lysis buffer (20 mmol/L Tris‐HCl pH7.4, 150 mmol/L NaCl, 0.1% sodiumdodecyl sulphate [SDS], 1% Sodium deoxycholate, 1% Triton, 1 mg Aprotinin, 1 mg Leupeptin) for 60 min on ice. Lysates were centrifuged at 2500*g* for 5 min. The protein concentration was determined by means of the Bradford protein assay (Bio‐Rad Labs, Richmond, CA) using bovine serum albumin as the standard. Twenty‐five micrograms of protein was resolved by electrophoresis through 12% polyacrylamide gels, was electrotransferred to a polyvinylidene difluoride filter (Millipore, Bedford, MA), and was then blotted with the first antibody. Following washing three times and second antibody incubation, the membranes were rinsed and the bound antibodies were detected using an enhanced chemiluminescence (ECL) detection system (Amersham, Buckinghamshire, UK). The primary antibodies used in this study were anti‐PAI‐1 polyclonal antibody (1:400 dilution; ab31280 Abcam, Cambridge, UK), anti‐MMP‐13 polyclonal antibody (1:400 dilution; ab39012 Abcam, Cambridge, UK), and anti‐*β*‐actin (1:2000; AC‐15; Sigma). The secondary antibodies used in this study were donkey anti‐goat IgG‐HRP (Santa Cruz Biotechnology, SantaCruz, CA, USA), goat anti‐rabbit IgG‐HRP (Santa Cruz Biotechnology), and anti‐mouse IgG‐HRP (MBL, Nagoya, Japan).

### Reporter assay

The 3′‐untranslated region (3′UTR) of PAI‐1 was polymerase chain reaction (PCR) amplified from genome DNA extracted from 143B cells. The PCR primers used to amplify the 3′UTR were as follows: forward, 5′‐CGCGTCTAGAGCTGGGGAAAGACGCCTTCATC‐3′ and reverse, 5′‐TTGTTCTAGAGGGCACGCATCTGACATTTC‐3′. The amplified 3′UTR was cloned downstream of the *Firefly* luciferase coding region in the pmirGLO plasmid (Promega, Madison, WI, USA). An mutant (Mut) construct was generated by mutating the seed region of the miR‐143 binding site by PCR mutagenesis. The 143B cells were seeded at a density of 2.0 × 10^4^ in 96‐well plates 24 h before transfection of the plasmids. The following day, 100 ng of reporter plasmid was cotransfected with 30 nmol/L of pre‐miR‐143 using the DharmaFECT transfection reagent (Thermo Scientific, Waltham, MA, USA). The cells were collected 24 h after transfection and assayed for luciferase activity using the Dual‐Luciferase‐Reporter Assay System (Promega) according to the manufacturer's protocol. Renilla luciferase was used for normalization. To assess the effect of the precursor miRNA on reporter activity, 100 ng of synthetic precursor miRNA (pre‐miR; Ambion, Invitrogen) was cotransfected with 100 ng of the reporter plasmid. All experiments were performed in triplicate.

### Cell proliferation and invasion assay

Twenty‐four hours after transfection, the cells were harvested and reseeded into a 96‐well plate. For the cell proliferation assay, the transfected cells were plated at a density of 2.5 × 10^3^ cells/well in a 96‐well plate and measured proliferation every 24 h using Cell Counting Kit‐8 according manufacture's protocol. The cell invasion assay was performed using the CytoSELECT 96‐Well Cell Invasion Assay kit (Cell Biolabs, San Diego, CA). Transfected cells were plated at a density of 2.5 × 10^5^ cells/well in 96‐well chambers. The manufacturer's protocol was followed (Cell Biolabs). Cells that had invaded the Matrigel were stained with hematoxylin and pores with stained cells and all pores were counted.

### Measurement of secreted MMP‐13 protein

Matrix metalloproteinase‐13 (MMP‐13) protein secreted into the conditioned media of cultured 143B cells was determined using the SensoLyte Plus 520 MMP‐13 Assay Kit (Anaspec, San Jose, CA). Briefly, the conditioned media were collected 48 h after transfection. In this assay, 50 *μ*L of 1 mmol/L p‐aminophenylmercuric acetate (APMA) treated medium was added to the collected conditioned media in each microplate well, followed by addition of the MMP‐13 substrate, the 5‐FAM/QXL520 fluorescence resonance energy transfer peptide. The fluorescence intensity representing pro‐MMP‐13 expression was measured at 485/510 nm wavelengths. All measurements were performed in triplicate.

### Immunohistochemistry

All tumors resected from mouse primary lesions at the right knee joint were fixed with 10% buffered formalin and embedded in paraffin. Thick sections of 3* μ*m were examined using immunohistochemistry. The sections were deparaffinized, and antigens were retrieved by autoclaving in 10 mmol/L citrate buffer (pH 6.0) at 121°C for 10 min. Endogenous peroxidase activity was blocked by immersing the slides in 0.6% hydrogen peroxide in methanol for 30 min. The sections were immunostained using a Histofine mouse staining kit (Nichirei, Tokyo, Japan). The primary antibodies used in this study were a mouse monoclonal antibody against human Ki‐67 (1:50; DAKO, Glostrup, Denmark), a goat polyclonal antibody against human PAI‐1 antigen (1:100; SEKISUI, Tokyo, Japan) and a rabbit polyclonal antibody against human MMP‐13 antigen (1:400; Abcam). Immunoreactions were visualized with diaminobenzidine and the sections were counterstained with hematoxylin.

### Animal model

All animal experiments were approved by the Institutional Animal Care and Use Committee of Tottori University (permit number: 13‐Y‐34). Five‐to six‐week‐old male athymic nude mice (CLEA Japan, Shizuoka, Japan) were anesthetized by exposure to 3% isoflurane on day 0 and subsequent days as indicated. On day 0, to generate the experimental model, the anesthetized animals were injected with 3.0 × 10^6^ 143B‐Luc cells into the knee of right hindlimb. Individual mice were injected with siRNA after complexation with in vivo‐jetPEI (Polyplus‐transfection, New York, NY) according to the manufacturer's protocol. Complexes of 12 *μ*g siRNA and in vivo‐jetPEI at a ratio of N/P (N = polymer amines, P = phosphate groups on pDNA) = 10 were prepared per mouse and were injected intratumorally in a volume of 100 *μ*L once every 3 days. The first injection was performed on day 1 post inoculation of 143B‐luc cells.

### RNA extraction and quantitative real‐time PCR of miRNAs

Total RNA was extracted from cell lines and clinical samples using the mirVana miRNA Isolation Kit (Ambion) according to the manufacture's protocol. MiR‐143‐specific complementary DNA was generated from 20 ng total RNA using the TaqMan MicroRNA RT kit (Applied Biosystems, Foster City, CA) and the miR‐143‐specific RT‐primer from the TaqMan Micro RNA Assay kit (Applied Biosystems). The expression levels of miR‐143 were measured using the miR‐143‐specific probe included with the TaqMan Micro RNA Assay kit on a Real‐Time PCR System 7300 with SDS software (Applied Biosystems).

### Statistical analyses

Statistical analyses were conducted using Student's *t*‐test for in vitro screening of cell invasion and proliferation, and also to evaluate lung metastasis in the in vivo assay. Welch's *t*‐test was used for miR‐143 expression analysis using clinical samples. *P *<* *0.05 was considered significant.

## Results

### PAI‐1 is a direct target gene of miR‐143 in 143B cells

**Figure 1:**
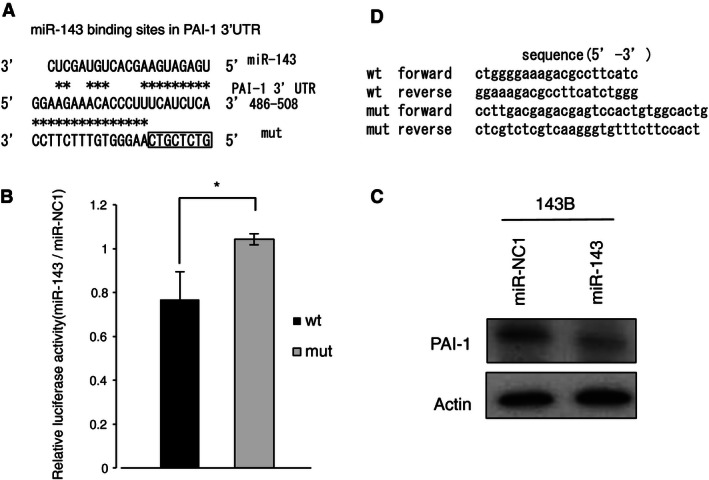
PAI‐1 is target gene of miR‐143 in 143B cells. (A) Alignment of the wild‐type PAI‐1 3′UTR (wt) and mutant PAI‐1 3′‐UTR (mut) with the miR‐143 binding site, displayed in the 3′–5′ orientation. (B) Reporter assay for analysis of the luciferase activity of the wt or mut luciferase reporter in 143B after transient transfection of miR‐143. The data were normalized to the control miR‐NC1. *P <* *0.05 (C) Western blot analysis of PAI‐1 expression in 143B after transient transfection of miR‐143 or the control miR‐NC1. PAI‐1 expression was quantified using ImageJ software and was normalized to *β*‐actin. Expression was calculated relative to that in miR‐NC1‐transfected cells. (D) The sequences of the wt and mut PAI‐1 3′UTR. PAI‐1, plasminogen activator inhibitor‐1; 3′UTR, 3′‐untranslated region.

To evaluate the function of miR‐143, it was important to determine whether PAI‐1 is a direct or indirect target gene of miR‐143 that might mediate the role of miR‐143 in invasion and metastasis. We found that the 3′UTR of PAI‐1 mRNA harbored a potential miR‐143 binding site (Fig. 1A). To confirm that PAI‐1 expression was directly inhibited by miR‐143, a dual‐luciferase reporter system was used. This assay showed that miR‐143 markedly suppressed the firefly luciferase reporter activity of the wild‐type PAI‐1 3′UTR, but did not change the activity of the mutant 3′UTR construct when transfected into 143B cells (Fig. 1B). Moreover, western blotting showed that miR‐143 also suppressed PAI‐1 protein expression (Fig. 1C). Thus, these results showed that miR‐143 directly bound to the 3′UTR region of PAI‐1 mRNA and suppressed PAI‐1 expression in 143B cells.

### Knockdown of PAI‐1 by siRNA transfection attenuated cell invasion ability but did not affect cell proliferation

**Figure 2:**
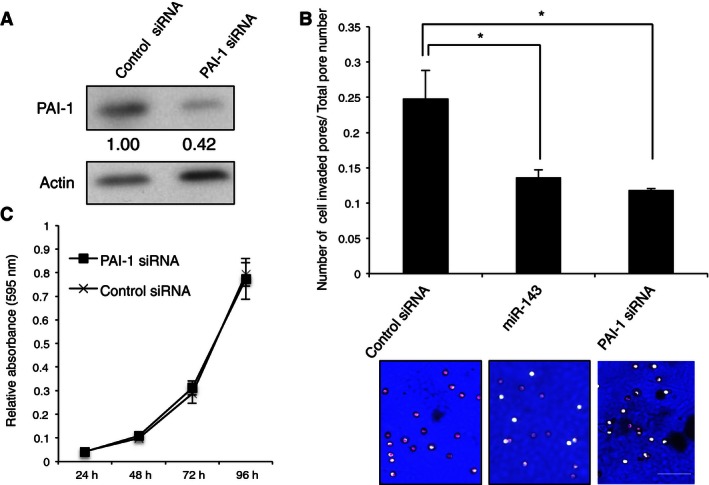
Plasminogen activator inhibitor‐1 (PAI‐1) regulates invasion without inhibiting proliferation of 143B cells. (A) Western blot analyses of PAI‐1 expression in 143B that were transfected with PAI‐1 or control siRNA. (B) Matrigel assay of the invasion of control siRNA, miR‐143, and PAI‐1 siRNA‐transfected cells. The ratio of the number of pores containing invading cells, as detected by staining with hematoxylin, to the total number of all pores is shown. *P <* *0.05. Representative pictures of membranes with invading cells, stained with hematoxylin, are provided at bottom. Scale bar, 50 *μ*m. (C) Proliferation assay after transient transfection of PAI or control siRNA into 143B cells. Relative absorbance (595 nm) to 24 h in 48, 72, and 96 h is shown. PAI‐1 expression was quantified using ImageJ software and was normalized to *β*‐actin. Expression is shown relative to that in control siRNA‐transfected cells.

We next examined the contribution of PAI‐1 to the ability of 143B cells to invade and proliferate in vitro using a PAI‐targeted siRNA. Western blotting showed that transfection of 143B cells with the PAI‐1 siRNA significantly decreased PAI‐1 protein expression (Fig. 2A). A Boyden Chamber assay with Matrigel demonstrated that 143B cells transfected with either PAI‐1 siRNA, or with miR‐143, displayed a significantly lower rate of invasion than cells transfected with control siRNA (Fig. 2B). In contrast, there was no difference in cell proliferation between PAI‐1 siRNA‐transfected and control siRNA‐transfected 143B cells (Fig. 2C). In addition, these siRNAs did not effect on migration activity in 143B cells (data not shown). These data indicate that PAI‐1 regulates the invasion, but not the proliferation and migration, in 143B cells.

### Suppression of lung metastasis of osteosarcoma cells by PAI‐1 siRNA in vivo

**Figure 3:**
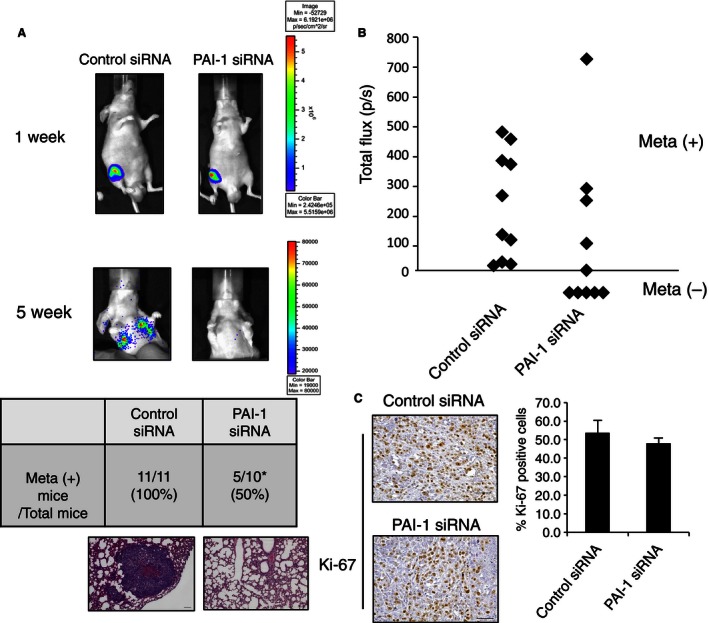
PAI‐1 siRNA regulates lung metastasis of 143B cells. (A) 143B‐luc cells were inoculated into the right knee of a model mouse. Representative bioluminescence at 1 and 5 weeks after inoculation as seen with the IVIS imager (top), and representative H&E staining of the lung at 5 weeks after inoculation (bottom) are shown. Scale bars, 100 *μ*m. The table in (A) shows the number of mice with lung metastasis (meta +) at 5 weeks over the total number of mice in the two groups. (B) Total Flux (photons per second, p/sec) measured in the obtained IVIS images of mice‐transfected control siRNA or PAI‐1 siRNA lung metastasis at 5 weeks after siRNA inoculation. (C) Representative histochemical staining of Ki‐67 in primary tumor cells from control and PAI‐1 siRNA‐transfected mice. The percentage of tumor cells that were positive for Ki‐67 was calculated by counting 10 visual fields at high magnification. Scale bars, 50 *μ*m. PAI‐1, plasminogen activator inhibitor‐1; IVIS, in vivo imaging system.

Next, we examined the role of PAI‐1 in lung metastasis of osteosarcoma cells in vivo using a previously reported spontaneous lung metastasis mouse model 13. The 143B‐luc cells (3.0 × 10^6^ cells) were inoculated into the right knee, and their location was immediately checked using an in vivo imaging system. After cell inoculation, 12 *μ*g PAI‐1 siRNA or control siRNA, complexed with in vivo‐jetPEI, was administered intratumorally to each group once every 3 days. After 1 week, a signal from firefly luciferase was detected only in the primary lesion, and no signals were detected in the pulmonary area in either group. At 5 weeks after inoculation, 11 of the 11 mice (100%) that were administered control siRNA displayed a luciferase signal from the pulmonary area suggesting lung metastasis; however, only 5 of the 10 mice (50%) that were administered PAI‐1 siRNA displayed a similar luciferase signal and the other five mice showed no signal in the pulmonary area. The difference in the number of control and siRNA‐administered mice with a luciferase signal in the pulmonary area was significant (Fig. 3A and B, *P* < 0.05). In addition, the lung metastasis of the osteosarcoma cells that was indicated by the luciferase signal was confirmed by histological analysis after autopsy. On the other hand, no differences were found in cell proliferation, as determined by Ki‐67 immunohistochemical staining, between primary tumor cells from the PAI‐1 siRNA group and those from the control siRNA group (Fig. 3C). Moreover, there were no differences in primary tumor size and volume (Fig. S1A and B). We confirmed PAI‐1 siRNA decreased PAI‐1 expression in primary tumor (Fig. S1C). The combined in vitro and in vivo data suggested that PAI‐1 promotes lung metastasis of osteosarcoma cells by facilitating tumor invasion activity, but not tumor cell proliferation.

### Knockdown of PAI‐1 downregulated MMP‐13 expression and secretion

**Figure 4:**
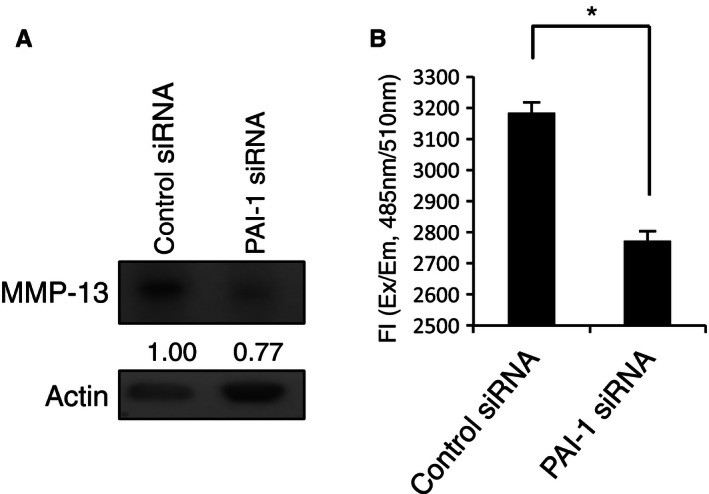
Knockdown of PAI‐1 suppressed MMP‐13 expression in 143B cells. (A) Western blot analysis of MMP‐13 expression in 143B cells that were transfected with PAI‐1 or control siRNA. MMP‐13 expression was quantified using ImageJ software and was normalized to *β*‐actin. Expression is shown relative to that in control siRNA‐transfected cells. (B) MMP‐13 levels in the conditioned media of the cells in (A) was determined using the Sensolyte MMP assay kit. The reaction was initiated by adding 100 *μ*L of the substrate solution. The fluorescence intensity of the reaction (Fl) was determined by calculation of the ratio of *λ* emission (Em) = 485 nm/*λ* excitation (Ex) = 520 nm. All assays were carried out in triplicate. *P <* *0.05. PAI‐1, plasminogen activator inhibitor‐1; MMP‐13, matrix metalloproteinase‐13.

To determine how PAI‐1 promotes invasion in 143B cells, we focused on its effects on the expression and secretion of MMP‐13. Western blotting showed that knockdown of PAI‐1 expression using siRNA caused a decrease in the expression level of the MMP‐13 protein versus control siRNA‐transfected cells (Fig. 4A). Moreover, the amount of MMP‐13 secreted from 143B cells transfected with PAI‐1 siRNA was significantly lower than that secreted from cells transfected with control siRNA (Fig. 4B, *P* < 0.05). These results suggested that knockdown of PAI‐1 reduces 143B cell invasion activity via downregulation of MMP‐13 expression and secretion. Then, we examined whether the MMP‐13 was one of target genes of miR‐143. Interestingly, we observed seed matches to residues of miR‐143 in MMP‐13 coding region between exon 7 and exon 8, and confirmed that MMP‐13, as well as PAI‐1, was one of the target genes of mir‐143 in 143B cell (Fig. S2), as strongly predicted in our previous report 13.

### Expression of miR‐143, PAI‐1, and MMP‐13 in clinical samples

**Figure 5:**
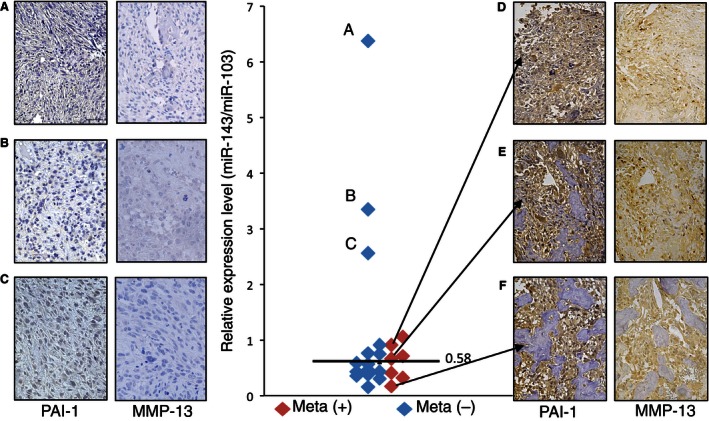
Expression of miR‐143, PAI‐1 and MMP‐13 in human osteosarcoma tissue samples. (A–F) PAI‐1 and MMP‐13 immunostained primary tumors of patients. Scale bars, 50 *μ*m. Central graph: The level of miR‐143 in 22 primary osteosarcoma specimens, including patients (A–F), was measured using real‐time reverse transcription (RT)‐PCR. Individual data points are the means of triplicate measurements from single RNA samples. The expression level of miR‐143 was normalized to that of miR‐103. Red symbols indicate lung metastasis‐positive (meta +) samples and blue symbols indicate lung metastasis‐negative (meta −) samples. The black bar indicates the mean value (0.58). PAI‐1, plasminogen activator inhibitor‐1; MMP‐13, matrix metalloproteinase‐13; PCR, polymerase chain reaction.

We then evaluated the expression of miR‐143 in human primary osteosarcomas in order to examine the correlation between miR‐143/PAI‐1 expression and lung metastasis. The expression level of miR‐143 in 22 biopsy samples of primary osteosarcoma without any metastases at first diagnosis was analyzed using real‐time RT‐PCR. The miR‐143 expression data were normalized to the mean expression level of miR‐103, which has been shown to be among the most stably expressed miRNAs in human tumor tissues 24. PAI‐1 expression was evaluated using immunohistochemistry. Few PAI‐1‐positive cells were observed in the three samples that had a high expression level of miR‐143. These samples were all lung metastasis‐negative, defined as showing no metastasis for at least >1 year after operation. The remaining 19 samples showed lower expression of miR‐143. Of these 19 samples, seven cases were lung metastasis‐positive. PAI‐1 and MMP‐13‐positive cells were frequently observed in these lung metastasis‐positive cases (Fig. 5). These data showed that low PAI‐1 and MMP‐13 expression was observed in osteosarcoma cases with higher miR‐143 expression, suggesting that downregulation of miR‐143 might lead to an increase in PAI‐1 expression.

**Figure 6:**
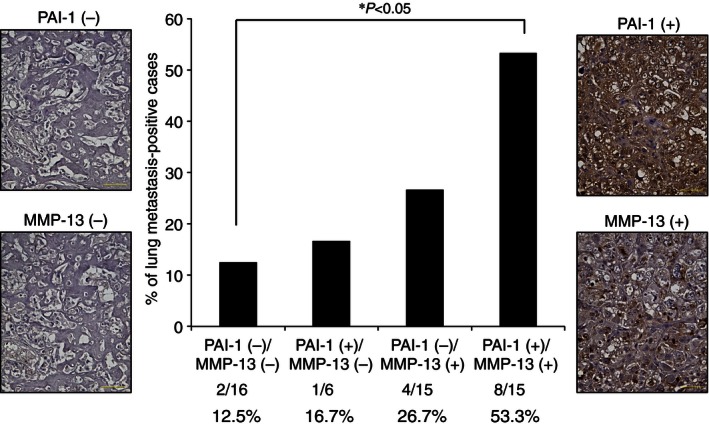
Expression of PAI‐1 and MMP‐13 in human osteosarcoma tissue samples. Fifty‐two primary osteosarcoma specimens, including the samples analyzed in Figure 5, were immunohistochemically analyzed for PAI‐1 and MMP‐13. Representative immunohistochemical staining of negative and positive cases are shown on the left and right hand sides, respectively, of the central graph. Tumors were considered positively stained if more than 10% of the cells stained positive for PAI‐1 or MMP‐13. The percentage of lung metastasis in tumors stained negative or positive for PAI‐1 and/or MMP‐13 is shown. There was no metastasis in any of samples at first diagnosis. The indicated *P*‐value was calculated using the chi‐squared test. *P <* *0.05. Scale bars, 50 *μ*m. PAI‐1, plasminogen activator inhibitor‐1; MMP‐13, matrix metalloproteinase‐13.

We finally immunohistochemically evaluated the expression of PAI‐1 and MMP‐13 in 52 human primary osteosarcoma samples in order to determine whether the expression pattern of the two proteins is correlated with lung metastasis of osteosarcoma cells. The 52 cases were classified into four groups: PAI‐1‐negative and MMP‐13‐negative (group 1, *n* = 16), PAI‐1‐positive and MMP‐13‐negative (group 2, *n* = 6), PAI‐1‐negative and MMP‐13‐positive (group 3, *n* = 15), and PAI‐1‐positive and MMP‐13‐positive (group 4, *n* = 15). As shown in Figure 6, the number of lung metastasis‐positive cases was significantly higher in PAI‐1 and MMP‐13 double‐positive cases (group 4) compared to any of the other groups (*P *<* *0.05). On the other hand, the differences between groups 1, 2, and 3 were not statistically significant. These results indicated that PAI‐1 and MMP‐13 double‐positive expression contributed significantly to the occurrence of lung metastasis of osteosarcoma.

## Discussion

In this report, we showed that PAI‐1 is a direct target gene of miR‐143 and regulates invasion in osteosarcoma. Knockdown of PAI‐1 by siRNA suppressed cell invasion activity without influencing cell proliferation, suggesting that PAI‐1 regulates invasiveness but not proliferation in human osteosarcoma cells. These data are supported by our previous report that miR‐143 both downregulates PAI‐1 and suppresses 143B‐cell invasion and metastasis without affecting cell proliferation 13. As shown in Figure 2C, the suppression of invasive activity by transfection of miR‐143 was the same as that of PAI‐1 siRNA, suggesting that the observed inhibition of invasion and metastasis by miR‐143 was mediated mainly via downregulation of PAI‐1 activity in 143B cells. In this study, immunohistochemical analyses of clinical osteosarcoma samples showed that the three cases that expressed the highest miR‐143 level showed few PAI‐1‐positive cells in the primary lesion and that all three of these cases were in the nonmetastatic group. On the other hand, strong or moderate staining of PAI‐1‐positive tumor cells was observed in the primary tumors of all the lung metastasis‐positive cases. These data suggested that downregulation of miR‐143 led to upregulation of PAI‐1 expression in human osteosarcoma cells and contributed to facilitation of lung metastasis from the primary lesion. Therefore, delivery of PAI‐1 siRNA into tumor cells could prevent lung metastasis of human osteosarcoma via suppression of PAI‐1 expression. Although higher expression of PAI‐1 in tumor cells contributes to cancer progression including metastasis in several malignancies 17, 18, 19, 20, 21, the mechanism by which PAI‐1 promotes the invasion and metastasis of osteosarcoma cells remains unknown. To address this mechanism, we focused on the expression and secretion of MMP‐13, which is a proteolytic enzyme that belongs to a large family of extracellular matrix‐degrading endopeptidases that are characterized by a zinc‐binding motif at their catalytic site 25. The overexpression of MMP‐13 has been documented in previous reports of breast cancer 26, squamous cell carcinoma of head and neck 27, lung cancer 28, malignant melanoma 29, colorectal cancer 30, oral squamous cell carcinoma 31, colorectal adenoma and carcinoma 32, and glioma 33. MMP‐13 expression in these human cancers was associated with cancer progression including cancer cell invasion and metastasis, diagnosis or poor outcome. Moreover, the expression of MMP‐13 has an important role in the pathogenesis of osteolysis by tumor cells in bone metastasis of human breast cancer 34 and prostate cancer 35. In addition, upregulation of MMP‐13 promotes the invasion of osteosarcoma cells 36 and higher expression of MMP‐13 is correlated with progression of human osteosarcoma 37. These studies suggest that MMP‐13 upregulation may have a similar role in the invasion of osteosarcoma cells at the primary lesion. This study showed that knockdown of PAI‐1 expression using siRNA‐mediated suppression resulted in a decrease in MMP‐13 expression and secretion in 143B cells, suggesting that MMP‐13 expression is regulated by PAI‐1 in osteosarcoma cells. However, the mechanism by which PAI‐1 regulates MMP‐13 expression in osteosarcoma cells has not been clarified. Jimenez et al. reported that the expression of MMP‐13 was upregulated by stimulation of human osteosarcoma cell lines with transforming growth factor beta (TGF‐beta), during which MMP‐13 transcription was directly promoted by the transcriptional activator Cbfa1 (core binding factor 1) 38. Furthermore, Oda et al. reported that PAI‐1 gene deficiency attenuates TGF‐beta1‐induced signaling in the renal fibrogenic response 39. These data suggest that transcriptional activation of genes that are upregulated by TGF‐beta is dependent on the PAI‐1 expression level in an inflammatory environment, which supports the possibility that suppression of MMP‐13 expression occurred by attenuation of TGF‐beta signaling following PAI‐1 knockdown in 143B cells. Thus, it is assumed that (1) the expression of both PAI‐1 and MMP‐13 genes strongly contributes to promotion of the invasion and metastasis, and (2) PAI‐1‐MMP‐13 axis is negatively regulated by miR‐143 in human osteosarcoma cells. Consistent with this hypothesis, expression of PAI‐1 and MMP‐13 were very poor in miR‐143 higher expressing osteosarcoma primary lesion (Fig. 5), and PAI‐1 and MMP‐13 double‐positive clinical samples (group 4) showed a significantly higher chance of lung metastasis than any of the other groups as shown in Figure 6. Further study is needed to clarify the mechanism by which PAI‐1 regulates MMP‐13 expression in human malignancies, at least in human osteosarcoma.

In conclusion, upregulation of PAI‐1 in human osteosarcoma cells is correlated with an increased risk of lung metastasis via an elevated level of MMP‐13 expression. Our findings indicated that PAI‐1 and MMP‐13 might be available metastasis‐prediction markers and also valuable target genes for preventing lung metastasis of osteosarcoma.

## Conflict of Interest

None declared.

## Supporting information


**Figure S1.** Weight of primary tumor after 5‐week treatment control siRNA or PAI‐1 siRNA (A). Quantification of tumor size in mm^3^ (B). Western blot analysis of PAI‐1 expression in primary tumor cells from control and PAI‐1 siRNA‐transfected mice. PAI‐1 expression was normalized to *γ*‐tubulin (C).

**Figure S2.** (A) Alignment of the wild‐type MMP‐13 coding region (wt) with the miR‐143 binding site, displayed in the 3′–5′ orientation twice and both binding sites are mutated in mutant MMP‐13 coding region (mut). (B) Reporter assay for analysis of the luciferase activity of the wt or mut luciferase reporter in 143B after transient transfection of miR‐143. The data were normalized to the control miR‐NC1. *P <* *0.05 (C) Western blot analysis of MMP‐13 expression in 143B after transient transfection of miR‐143 or the control miR‐NC1. MMP‐13 expression was quantified using ImageJ software and was normalized to *β*‐actin. Expression was calculated relative to that in miR‐NC1‐transfected cells. (D) The sequences of the wt and mut MMP‐13 coding region.

## References

[1] Wada, T. , K. Isu , N. Takeda , M. Usui , S. Ishii , and S. Yamawaki . 1996 A preliminary report of neoadjuvant chemotherapy NSH‐7 study in osteosarcoma: preoperative salvage chemotherapy based on clinical tumor response and the use of granulocyte colony‐stimulating factor. Oncology 53:221–227. 8643225 10.1159/000227564

[2] Kempf‐Bielack, B. , S. S. Bielack , H. Jurgens , D. Branscheid , W. E. Berdel , G. U. Exner , et al. 2005 Osteosarcoma relapse after combined modality therapy: an analysis of unselected patients in the Cooperative Osteosarcoma Study Group (COSS). J. Clin. Oncol. 23:559–568. 15659502 10.1200/JCO.2005.04.063

[3] Pasquinelli, A. E. , B. J. Reinhart , F. Slack , M. Q. Martindale , M. I. Kuroda , B. Maller , et al. 2000 Conservation of the sequence and temporal expression of let‐7 heterochronic regulatory RNA. Nature 408:86–89. 11081512 10.1038/35040556

[4] Bartel, D. P. 2004 MicroRNAs: genomics, biogenesis, mechanism, and function. Cell 116:281–297. 14744438 10.1016/s0092-8674(04)00045-5

[5] Lewis, B. P. , C. B. Burge , and D. P. Bartel . 2005 Conserved seed pairing, often flanked by adenosines, indicates that thousands of human genes are microRNA targets. Cell 120:15–20. 15652477 10.1016/j.cell.2004.12.035

[6] Lu, J. , G. Getz , E. A. Miska , E. Alvarez‐Saavedra , J. Lamb , D. Peck , et al. 2005 MicroRNA expression profiles classify human cancers. Nature 435:834–838. 15944708 10.1038/nature03702

[7] Volinia, S. , G. A. Calin , C. G. Liu , S. Ambs , A. Cimmino , F. Petrocca , et al. 2006 A microRNA expression signature of human solid tumors defines cancer gene targets. Proc. Natl. Acad. Sci. USA 103:2257–2261. 16461460 10.1073/pnas.0510565103 PMC1413718

[8] Calin, G. A. , M. Ferracin , A. Cimmino , G. Di Leva , M. Shimizu , S. E. Wojcik , et al. 2005 A MicroRNA signature associated with prognosis and progression in chronic lymphocytic leukemia. N. Engl. J. Med. 353:1793–1801. 16251535 10.1056/NEJMoa050995

[9] Jiang, J. , Y. Gusev , I. Aderca , T. A. Mettler , D. M. Nagorney , D. J. Brackett , et al. 2008 Association of MicroRNA expression in hepatocellular carcinomas with hepatitis infection, cirrhosis, and patient survival. Clin. Cancer Res. 14:419–427. 18223217 10.1158/1078-0432.CCR-07-0523 PMC2755230

[10] Asangani, I. A. , S. A. K. Rasheed , D. A. Nikolova , J. H. Leupold , N. H. Colburn , S. Post , et al. 2008 MicroRNA‐21 (miR‐21) post‐transcriptionally downregulates tumor suppressor Pdcd4 and stimulates invasion, intravasation and metastasis in colorectal cancer. Oncogene 27:2128–2136. 17968323 10.1038/sj.onc.1210856

[11] Valastyan, S. , F. Reinhardt , N. Benaich , D. Calogrias , A. M. Szász , Z. C. Wang , et al. 2009 A pleiotropically acting microRNA, miR‐31, inhibits breast cancer metastasis. Cell 137:1032–1046. 19524507 10.1016/j.cell.2009.03.047 PMC2766609

[12] Cimmino, A. , G. A. Calin , M. Fabbri , M. V. Iorio , M. Ferracin , M. Shimizu , et al. 2005 miR‐15 and miR‐16 induce apoptosis by targeting BCL2. Proc. Natl. Acad. Sci. USA 102:13944–13949. 16166262 10.1073/pnas.0506654102 PMC1236577

[13] Osaki, M. , F. Takeshita , Y. Sugimoto , N. Kosaka , Y. Yamamoto , Y. Yoshioka , et al. 2011 MicroRNA‐143 regulates human osteosarcoma metastasis by regulating matrix metalloprotease‐13 expression. Mol. Ther. 19:1123–1130. 21427707 10.1038/mt.2011.53 PMC3129798

[14] Dawson, S. , and A. Henney . 1992 The status of PAI‐1 as a risk factor for arterial and thrombotic disease: a review. Atherosclerosis 95:105–117. 1418086 10.1016/0021-9150(92)90014-8

[15] Ferroni, P. , M. Roselli , I. Portarena , V. Formica , S. Riondino , and F. LA Farina . 2014 Plasma plasminogen activator inhibitor‐1 (PAI‐1) levels in breast cancer—relationship with clinical outcome. Anticancer Res. 34:1153–1162. 24596353

[16] Ren, F. , H. Shi , G. Zhang , and R. Zhang . 2013 Expression of deleted in liver cancer 1 and plasminogen activator inhibitor 1 protein in ovarian carcinoma and their clinical significance. J. Exp. Clin. Cancer Res. 32:60. 23988121 10.1186/1756-9966-32-60 PMC3848092

[17] Santhekadur, P. K. , M. Akiel , L. Emdad , R. Gredler , J. Srivastava , D. Rajasekaran , et al. 2014 Staphylococcal nuclease domain containing‐1 (SND1) promotes migration and invasion via angiotensin 2 type 1 receptor (AT1R) and TGF‐beta signaling. FEBS Open Bio 4:353–361. 10.1016/j.fob.2014.03.012 PMC4050181 24918049

[18] Bajou, K. , A. Noel , R. D. Gerard , V. Masson , N. Brunner , C. Holst‐Hansen , et al. 1998 Absence of host plasminogen activator inhibitor 1 prevents cancer invasion and vascularization. Nat. Med. 4:923–928. 9701244 10.1038/nm0898-923

[19] Isogai, C. , W. E. Laug , H. Simada , P. J. Declerck , M. F. Stins , D. L. Durden , et al. 2001 Plasminogen activator inhibitor‐1 promotes angiogenesis by stimulating endothelial cell migration toward fibronectin. Cancer Res. 61:5587–5594. 11454712

[20] Bajou, K. , C. Mallard , M. Jost , R. H. Lijnen , A. Gils , P. Declerck , et al. 2004 Host‐derived plasminogen activator inhibitor‐1 (PAI‐1) concentration is critical for in vivo tumoral angiogenesis and growth. Oncogene 23:6986–6990. 15286708 10.1038/sj.onc.1207859

[21] Yan, L. , and L. C. Demars . 2014 Effect of a high‐fat diet on spontaneous metastasis of Lewis lung carcinoma in plasminogen activator inhibitor‐1 deficient and wild‐type mice. PLoS One 9:e110869. 25356654 10.1371/journal.pone.0110869 PMC4214820

[22] Meryet‐Figuières, M. , S. Resina , C. Lavigne , G. Barlovatz‐Meimon , B. Lebleu , and A. R. Thierry . 2007 Inhibition of PAI‐1 expression in breast cancer carcinoma cells by siRNA at nanomolar range. Biochimie 89:1228–1233. 17509745 10.1016/j.biochi.2007.03.017

[23] Suzuki, Y. , H. Mogami , H. Ihara , and T. Urano . 2009 Unique secretory dynamics of tissue plasminogen activator and its modulation by plasminogen activator inhibitor‐1 in vascular endothelial cells. Blood 113:470–478. 18922856 10.1182/blood-2008-03-144279

[24] Peltier, H. J. , and G. J. Latham . 2008 Normalization of microRNA expression levels in quantitative RT‐PCR assays: identification of suitable reference RNA targets in normal and cancerous human solid tissues. RNA 14:844–852. 18375788 10.1261/rna.939908 PMC2327352

[25] Leeman, M. F. , S. Curran , and G. I. Murray . 2002 The structure, regulation, and function of human matrix metalloproteinase‐13. Crit. Rev. Biochem. Mol. Biol. 37:149–166. 12139441 10.1080/10409230290771483

[26] Chang, H. J. , M. J. Yang , Y. H. Yang , M. F. Hou , E. J. Hsueh , and S. R. Lin . 2009 MMP13 is potentially a new tumor marker for breast cancer diagnosis. Oncol. Rep. 22:1119–1127. 19787229 10.3892/or_00000544

[27] Stokes, A. , J. Joutsa , R. Ala‐Aho , M. Pitchers , C. J. Pennington , C. Martin , et al. 2010 Expression profiles and clinical correlations of degradome components in the tumor microenvironment of head and neck squamous cell carcinoma. Clin. Cancer Res. 16:2022–2035. 20305301 10.1158/1078-0432.CCR-09-2525

[28] Hsu, C. P. , G. H. Shen , and J. L. Ko . 2006 Matrix metalloproteinase‐13 expression is associated with bone marrow microenvironment and prognosis in non‐small cell lung cancer. Lung Cancer 52:349–357. 16569461 10.1016/j.lungcan.2006.01.011

[29] Corte, M. D. , L. O. Gonzalez , M. G. Corte , I. Quintela , I. Pidal , M. Bongera , et al. 2005 Collagenase‐3 (MMP‐13) expression in cutaneous malignant melanoma. Int. J. Biol. Markers 20:242–248. 16398406 10.1177/172460080502000407

[30] Huang, M. Y. , H. J. Chang , F. Y. Chung , M. J. Yang , Y. H. Yang , J. Y. Wang , et al. 2010 MMP13 is a potential prognostic marker for colorectal cancer. Oncol. Rep. 24:1241–1247. 20878116

[31] Vincent‐Chong, V. K. , I. Salahshourifar , L. P. Karen‐Ng , M. Y. Siow , T. G. Kallarakkal , A. Ramanathan , et al. 2014 Overexpression of MMP‐13 is associated with clinical outcomes and poor prognosis in oral squamous cell carcinoma. Sci. World J. 2014:897523. 10.1155/2014/897523 PMC4226172 25401159

[32] Foda, A. A. , A. K. El‐Hawary , and A. Abdel‐Aziz . 2014 Matrix metalloproteinase‐13 expression in the progression of colorectal adenoma to carcinoma: matrix metalloproteinase‐13 expression in the colorectal adenoma and carcinoma. Tumour Biol. 35:5653–5658. 24563279 10.1007/s13277-014-1748-9

[33] Wang, J. , Y. Li , J. Wang , C. Li , K. Yu , and Q. Wang . 2012 Increased expression of matrix metalloproteinase‐13 in glioma is associated with poor overall survival of patients. Med. Oncol. 29:2432–2437. 22351249 10.1007/s12032-012-0181-4

[34] Nannuru, K. C. , M. Futakuchi , M. L. Varney , T. M. Vincent , E. G. Marcusson , and R. K. Singh . 2010 Matrix metalloproteinase (MMP)‐13 regulates mammary tumor‐induced osteolysis by activating MMP9 and transforming growth factor‐beta signaling at the tumor‐bone interface. Cancer Res. 70:3494–3504. 20406980 10.1158/0008-5472.CAN-09-3251 PMC2862120

[35] Akech, J. , J. J. Wixted , K. Bedard , M. van der Deen , S. Hussain , T. A. Guise , et al. 2010 Runx2 association with progression of prostate cancer in patients: mechanisms mediating bone osteolysis and osteoblastic metastatic lesions. Oncogene 29:811–821. 19915614 10.1038/onc.2009.389 PMC2820596

[36] Zhou, Y. , Z. Hu , N. Li , and R. Jiang . 2015 Interleukin‐32 stimulates osteosarcoma cell invasion and motility via AKT pathway‐mediated MMP‐13 expression. Int. J. Mol. Med. 35:1729–1733. 25846944 10.3892/ijmm.2015.2159

[37] Korpi, J. T. , J. Hagström , N. Lehtonen , J. Parkkinen , T. Sorsa , T. Salo , et al. 2011 Expression of matrix metalloproteinases‐2, ‐8, ‐13, ‐26, and tissue inhibitors of metalloproteinase‐1 in human osteosarcoma. Surg. Oncol. 20:e18–e22. 20880700 10.1016/j.suronc.2010.08.004

[38] Jimenez, M. J. , M. Balbin , J. M. Lopez , J. Alvarez , T. Komori , and C. LoPez‐Otin . 1999 Collagenase 3 is a target of Cbfa1 a transcription factor of the *runt* gene family involved in bone formation. Mol. Cell. Biol. 19:4431–4442. 10330183 10.1128/mcb.19.6.4431 PMC104402

[39] Oda, T. , Y. O. Jung , H. S. Kim , X. Cai , J. M. López‐Guisa , Y. Ikeda , et al. 2001 PAI‐1 deficiency attenuates the fibrogenic response to ureteral obstruction. Kidney Int. 30:587–596. 11473641 10.1046/j.1523-1755.2001.030002587.x

