# Supplementary figures and images for: PAI‐1, a target gene of miR‐143, regulates invasion and metastasis by upregulating MMP‐13 expression of human osteosarcoma
Source: Cancer Med. 2016 Jan 28;5(5):892–902. doi: 10.1002/cam4.651 (PMC4864819; doi:10.1002/cam4.651)

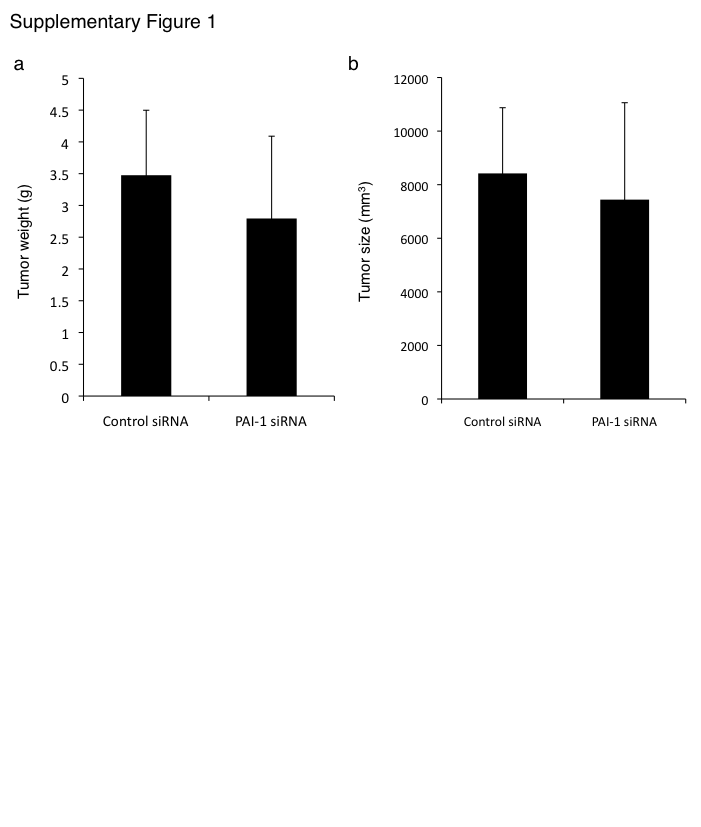

Supplement: Supplementary file 1 — Figure S1. Weight of primary tumor after 5‐week treatment control siRNA or PAI‐1 siRNA (A). Quantification of tumor size in mm3 (B). Western blot analysis of PAI‐1 expression in primary tumor cells from control and PAI‐1 siRNA‐transfected mice. PAI‐1 expression was normalized to γ‐tubulin (C). [file CAM4-5-892-s001.tif]

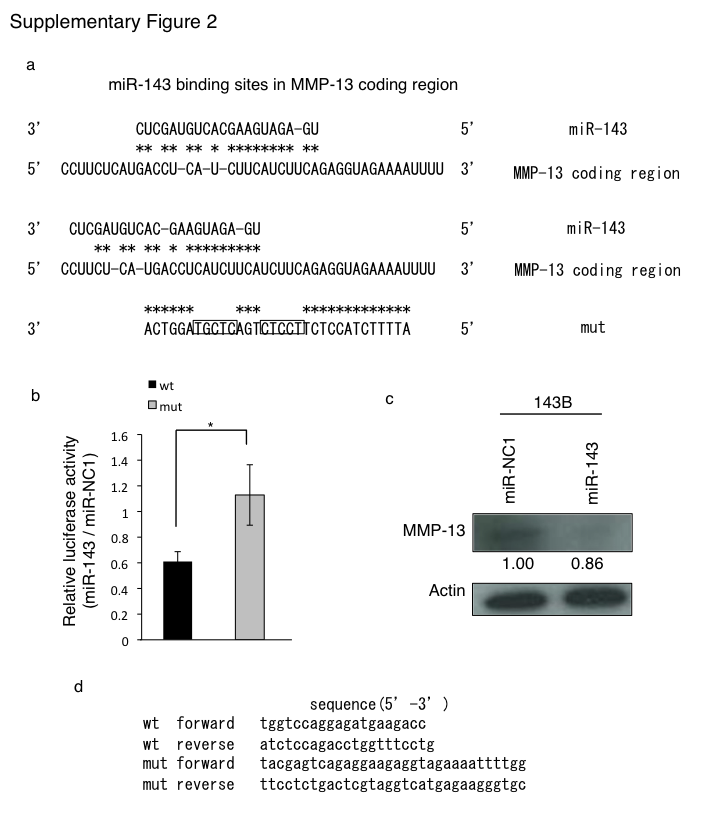

Supplement: Supplementary file 2 — Figure S2. (A) Alignment of the wild‐type MMP‐13 coding region (wt) with the miR‐143 binding site, displayed in the 3′–5′ orientation twice and both binding sites are mutated in mutant MMP‐13 coding region (mut). (B) Reporter assay for analysis of the luciferase activity of the wt or mut luciferase reporter in 143B after transient transfection of miR‐143. The data were normalized to the control miR‐NC1. *P < 0.05 (C) Western blot analysis of MMP‐13 expression in 143B after transient transfection of miR‐143 or the control miR‐NC1. MMP‐13 expression was quantified using ImageJ software and was normalized to β‐actin. Expression was calculated relative to that in miR‐NC1‐transfected cells. (D) The sequences of the wt and mut MMP‐13 coding region. [file CAM4-5-892-s002.tif]
